# Making European performance and impact assessment frameworks glocal

**DOI:** 10.12688/f1000research.108804.1

**Published:** 2022-03-04

**Authors:** Ana M.P. Melo, Sofia Oliveira, Jorge S. Oliveira, Corinne S. Martin, Ricardo B. Leite

**Affiliations:** 1 BioData.pt - Portuguese Infrastructure of Biological Data, Oeiras, Portugal; 2 INESC ID - Instituto Nacional de Engenharias de Sistemas e Computadores - Investigação e Desenvolvimento, Lisboa, Portugal; 3 Instituto Gulbenkian de Ciência, Oeiras, Portugal; 4 ELIXIR Hub, Wellcome Genome Campus, Hinxton, Cambridge, CB10 1SD, UK

**Keywords:** biological data, FAIR, research management, open science, performance and impact indicators, research infrastructures, socio-economic impact

## Abstract

Sustainability of research infrastructures (RIs) is a big challenge for funders, stakeholders and operators, and the development and adoption of adequate management tools is a major concern, namely tools for monitoring and evaluating their performance and impact. BioData.pt is the Portuguese Infrastructure of Biological and Portuguese node of the European Strategy Forum on Research Infrastructures "Landmark" ELIXIR. The foundations of this national research infrastructure were laid under the “Building BioData.pt” project, for four years. During this period, performance and impact indicators were collected and analysed under the light of international guidelines for assessing the performance and impact of European research infrastructures produced by the European Strategy Forum on Research Infrastructures, the Organisation for Economic Co-operation and Development and the EU-funded RI-PATHS project. The exercise shared herein showed that these frameworks can be adopted by national RIs, with the necessary adaptations, namely to reflect the national landscape and specificity of activities, and can be powerful tools in supporting the management of RIs.

“Not everything that counts can be counted, and not everything that can be counted, counts”. Albert Einstein, Theoretical physicist and Nobel Prize winner

## Introduction

The long-term sustainability of Research Infrastructures (RIs) is of great importance to the European Strategic Forum on Research Infrastructures (ESFRI) and the European Union more broadly, as shown by calls for RIs to demonstrate their economic and wider benefit to society. ^1^ For the Organisation for Economic Co-operation and Development (OECD), sustainability is also a major concern as RIs represent an increasingly large share of research investment by national governments. ^2^ As a result, recent years have seen the emergence of a number of frameworks ( ESFRI, OECD and that developed by the EU-funded RI-PATHS project) to guide RIs in their journeys to demonstrate performance and impact, going beyond simply scientific impact, and considering public value more generally.

### Motivation

BioData.pt is the Portuguese Infrastructure of Biological Data and the Portuguese node of ELIXIR. In this study, the goal was to consider three frameworks for impact and performance evaluation (ESFRI, OECD and RI-PATHS) in relation to existing indicators maintained by the BioData.pt project, from now on referred to as “Building BioData.pt”, funded via the Portuguese state budget and European structural funds. From this, we expected to gain a more systematic, structured, and deeper understanding of the performance and impact of this national-level RI, and to use this new knowledge to inform its further development towards long-term sustainability. Beyond the qualitative assessment itself, this exercise aimed to document a process that may help national nodes of distributed European RIs to understand the impact of their activities, to inform stakeholders, policy makers and funders, as well as to improve their operations and increase their visibility, and overall prestige. In addition, it aimed to assess the relevance and adequacy of the indicators used by BioData.pt. It is expected that our findings, including lessons learned, will be useful to other similar public-funded RIs, which are often working with limited resources and do not have the means to fund repeated impact evaluations by specialized consultancies.

### Organization and historical path

**Figure 1.**
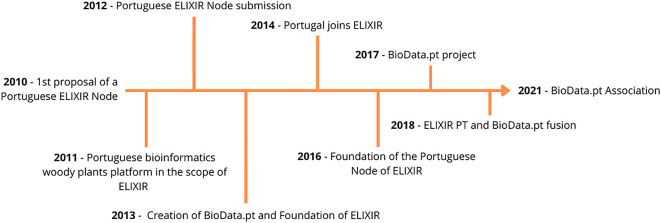
Historical path of the Portuguese Node of ELIXIR. In 2010, a Portuguese researcher from IGC (Instituto Gulbenkian de Ciência) at the European Bioinformatics Institute was challenged to participate in the onset of ELIXIR and create the Portuguese Node of this, yet to be, research infrastructure. In 2011, a first step was done, by creating the Portuguese bioinformatics woody plants platform followed by the creation of BioData.pt – Portuguese Biological Data Network, to operate the Portuguese Node of ELIXIR (ELIXIR PT), in 2013. In the same year, the ELIXIR, an intergovernmental organisation bringing together life science resources from across Europe, namely, databases, software tools, training materials, cloud storage and supercomputers, was founded. In 2014, the Foundation for Science and Technology, on behalf of the Portuguese Government, signed the ELIXIR Consortium Agreement, by which Portugal adhered to ELIXIR and, in 2016, a collaboration agreement between INESC-ID (Instituto de Engenharia de Sistemas e Computadores, Investigação e Desenvolvimento) and ELIXIR was signed, creating the Portuguese Node of this pan-European infrastructure. The merger between BioData.pt and ELIXIR PT was formalized in 2018 and, very recently, already in 2021, the BioData.pt Association was founded as the legal entity of this RI.

Although BioData.pt is the Portuguese Infrastructure of Biological Data and the Portuguese Node of the ESFRI Landmark ELIXIR (a pan-European research infrastructure for life science data, Ref. 3), it relies on fixed-term project funds to operate. A historical note of BioData.pt and the Portuguese Node of ELIXIR is depicted in Figure 1.

Since 2017, the development and early operation of BioData.pt have been funded through a €2.7M project grant to last for four years, to lay the foundation of this RI. The “Building BioData.pt” project involved 11 beneficiaries and 81 participants, many of which providing in-kind expertise on computing, bioinformatics and data management to a range of end-users, as well as building and operating the infrastructure itself. Of the project budget, 33% was used to hire human resources and 57% to purchase equipment, mostly computing resources to assemble the nationally distributed computing infrastructure. The remainder was used in the adaptation of buildings to host the computing infrastructure or to deliver training. “Building BioData.pt” was structured as shown in Table 1.

**Table 1.** Working groups (WG) of the “Building BioData.pt” project, which was instrumental to the creation and early operation of the BioData.pt infrastructure.

Research Communities	Support to Research Communities	Industry Engagement
WG1 - Plants	WG6 - Common Infrastructure	WG8 - Industry & Entrepreneurship
WG2 - Marine Resources	WG7 - Training	
WG3 - Systems biology	WG3 - Systems biology	
WG4 - Neurosciences	WG10 - Project Management & Dissemination	
WG5 - Yeastract		

## Methods

**Figure 2.**
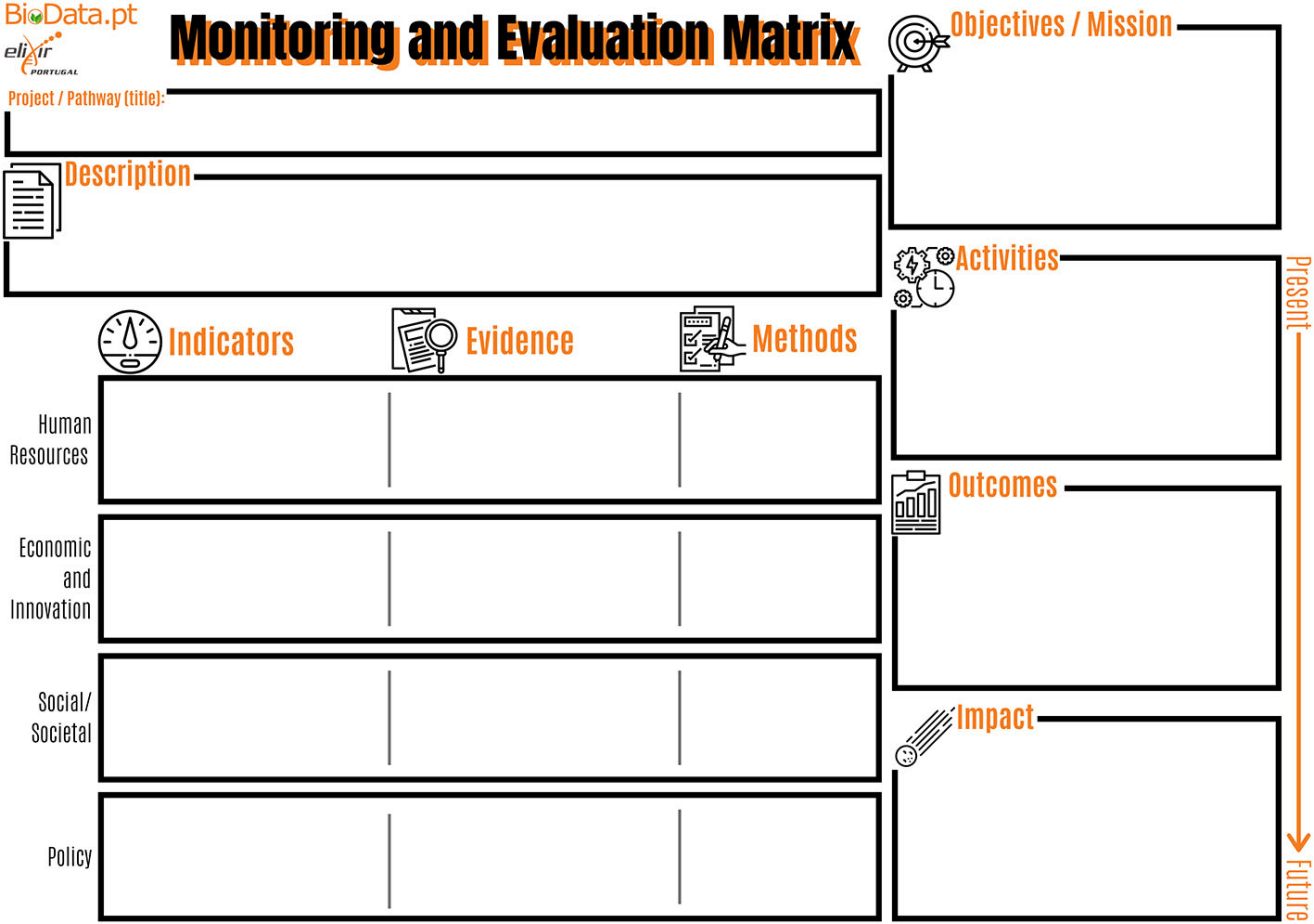
BioData.pt Monitoring and Evaluation Matrix. This matrix, internally developed during the BioData.pt project, was used to understand and document project working groups in terms of activities, outcomes and impact.

Based on the simplified RI-PATHS approach, which classifies indicator types in activity (concrete activities to be carried out within the scope of the project, visible to the public and under full control of the organization), outcome (short-term direct results of each activity, not under direct control of the organization) and impact (transformative effects of the activity on its target audience and beyond, in the mid to long term), ^4^ a “BioData.pt Monitoring and Evaluation Matrix” (B-MEM) ( Figure 2, Ref. 5) and supporting guidelines were developed to facilitate the onset of evaluation processes, by the BioData.pt infrastructure. This simple and participatory approach allowed project managers to view and document, for the first time, the 10 working groups (or work packages) ( Table 1) of “Building BioData.pt” in terms of activities, outcomes and impact, and align these with the overarching objectives of the project. In a second stage, the existing “Building BioData.pt” indicators, which had been maintained during the four years of grant execution, were categorized as activity, outcome and impact indicators, and assigned to the most relevant RI-PATHS impact area.

Each existing “Building BioData.pt” indicator, which could be quantitative or qualitative by nature, was documented and cross-checked against those compiled by the RI-PATHS project, and the OECD and ESFRI frameworks.

Finally, the “Building BioData.pt” indicators were assessed against several sets of strategic objectives relevant to research infrastructures and their work: those of BioData.pt (as an organization), those of the current ELIXIR Scientific Programme, ^6^ those of the Portuguese National Roadmap of Research Infrastructures, ^7^ those of EOSC ^8^ and, finally, those of the United Nations Sustainable Development Goals. ^9^ This was done to gain insights into the relevance of BioData.pt in these broader contexts.

A visualization method was used to better document the relevance of the retrieved indicators, considering the national context, and the global environment of BioData.pt mission. A stacked column chart was generated entirely using the Rstudio software version 2021.09.0.351 and *ggplot2* version 3.3.5, ^10^ specifically using the ggplot() function, due to the complexity of the graphic. The *fct_reorder()* function of *forcats* version 0.5.1 was used to reorder indicators according to their relevance, in decreasing order.

## Implementation and impact analysis

The first step was the description of the “Building BioData.pt” project working groups, in terms of activities, outcomes and impact, and their alignment with its two overarching objectives, using the BioData.pt Impact Assessment Matrix (B-MEM) and the respective guidelines. Working groups (WG) 1 to 5 were dedicated to domain specific activities which, overall, aimed to build the BioData.pt Research Communities, and their expected outcomes were data curation, integration and availability for domain-specific areas with impact in enhancing the quality of scientific research by promoting the FAIR principles. The global activity of WGs 6, 7, 9 and 10 was to set up the Support to Research Communities, having three outcome/impact lines: 1) a fully available computational infrastructure for data analysis/better and more extensive computing resources; 2) promotion of capacity-building in bioinformatics and research data management in the national research community/improved research efficiency & effectiveness and human capital; and 3) overall management of RI and operation of the Portuguese Node of ELIXIR/strengthened RI long-term sustainability. Both “Research Communities” and “Support to Research Communities” were developed under the objective “Strengthening research, technological development and innovation”. The second objective “Enhancing research knowledge transfer from academy to industry” was served by the activity Industry & Entrepreneurship, developed by WG8, delivering promotion of knowledge transfer to industry to bring an added value to companies’ data and an industrial ecosystem of companies aware/beneficiary of bioinformatics and research data management as outcome and impact, respectively.

Yet with the help of the B-MEM, the “Building BioData.pt” project and Infrastructure reports were mined for indicators that would relate to those compiled by the ESFRI, OECD and RI-PATHS frameworks. Table 2 shows one example of this implementation, performed for the activity “Research Communities”, in relation to RI-PATHS. In detail, for this activity, we were able to find indicators of two types, activity and outcome, distinctly distributed by three of four impact areas, Human Resources, Economic and Innovation and Societal/Social. For each type of indicator identified, the indicator itself, the evidence for confirmation and method for description is listed. For instance, under the objective “Strengthening research, technological development and innovation”, in the activity “Research Communities”, the activity indicator *Number of publications* is identified by direct evidence (e.g., from Web of Science) and the method for collecting evidence is counting the number of publications. The full dataset of BioData.pt project indicators classified following the RI-PATHS approach can be accessed. ^11^

**Table 2.** Example of primary data collected with the B-MEM, for “Building BioData.pt” activity *Research Communities.* B-MEM follows the RI-PATHS approach, thus segmenting indicator types in Activity, Outcome or Impact, and requiring further description of the Evidence and Method used for indicator collection. WG (Working Group) 1 – Plants, WG2 – Marine Resources, WG3 – Systems biology, WG4 – Neurosciences, WG5 – Yeastract.

**Project:**		BioData.pt - Portuguese Infrastructure of Biological Data	
**Description:**		BioData.pt is a research infrastructure that brings computing, bioinformatics and data management to the field of life sciences (health, biotechnology, marine and agroforest to enhance data intensive research)	
**Objectives/Mission**		O1. Strengthening research, technological development and innovation	
**Activity**		Research Communities (WG1, 2, 3, 4, 5)	
**Outcome**		Data curation, integration and availability for domain-specific areas	
**Impact**		Enhance the quality of scientific research by promoting FAIR principles	
**Human Resources**	**Type of indicator**	Activity	Outcome
	**Indicator**	Number of publications	Citations for publications
	**Evidence**	Direct	Direct
	**Method**	Counting	Counting
**Economic and innovation**	**Type of indicator**	Activity	Activity
	**Indicator**	Number of computing applications developed	Research results fed into shared datasets/repositories
	**Evidence**	Direct	External report
	**Method**	Counting	Liaison with service accountables
**Social/Societal**	**Type of indicator**	Outcome	
	**Indicator**	Use of open data	
	**Evidence**	Statistics of CorkOak DB portal	
	**Method**	Deduction	
**Policy**	**Type of indicator**	No indicator found	
	**Indicator**		
	**Evidence**		
	**Method**		

This exercise was repeated using the impact assessment frameworks of ESFRI and OECD. These approaches cover more explicitly the so-called scientific impact, of which some indicators, like *Number of publications* and *Number of Citations*, are also covered in the Human Resources area of the RI-PATHS approach.

A summary of RI-PATHS’ indicator types produced by “Building BioData.pt” ( Table 3) shows that the different activities of the project, “Research Communities”, “Support to Research Communities” and “Industry and Entrepreneurship”, encompassing its 10 working groups had impact in all four RI-PATHS areas, namely, Human Resources, Economic and Innovation, Social/Societal and Policy, the working groups that provide “Support to Research Communities” having the broader socio-economic impact.

**Table 3.** “Building BioData.pt” indicators found in the RI-PATHS project indicators and impact areas. A- activity; O- outcome; I- impact; Tick – indicator or proxy found; n/a – not applicable. WG (Working Group) 1 – Plants, WG2 – Marine Resources, WG3 – Systems biology, WG4 – Neurosciences, WG5 – Yeastract, WG6 – Common Infrastructure, WG7 – Training, WG8 – Industry & Entrepreneurship, WG9 – Bioinformatics Support and WG10 – Project Management & Dissemination.

Impact area	Indicator type	BioData.pt Infrastructure Objectives	Strengthening research, technological development and innovation		Enhancing research knowledge transfer from academy to industry
		BioData.pt Project Working Groups	Research Communities (WG1, 2, 3, 4, 5)	Support to Research Communities (WG6, 7, 9, 10)	Industry & Entrepreneurship (WG8)
**Human Resources**	A	Number of publications	√	n/a	n/a
	A	Number of events organized by RI (nr. participants)	n/a	√	n/a
	A	Number of researchers working in improved research infrastructures	n/a	√	n/a
	A	Number of long-term higher education training programmes	n/a	√	n/a
	O	Satisfaction of people trained	n/a	√	n/a
	O	Citations for publications	√	n/a	n/a
	I	Increased prestige as training facility	n/a	√	n/a
	I	Improvement of HRST	n/a	√	n/a
**Economic and innovation**	A	Number of computing applications developed	√	n/a	n/a
	A	Research results fed into shared datasets/repositories	√	n/a	√
	A	Number of collaborations with industry	n/a	n/a	√
	O	Uptake of accessible datasets/instruments/tools outside RI	n/a	√	√
	O	Firms using an RI technology	n/a	n/a	√
	I	Corporate efficiency gains through use/application of RI data	n/a	n/a	√
**Social/Societal**	A	People reached and engaged in outreach activities	n/a	√	√
	A	Visitors on website and followers on social media	n/a	√	n/a
	A	Number of scientific users	n/a	√	n/a
	O	Satisfaction of scientific users	n/a	√	n/a
	O	Use of open data	√	n/a	n/a
	I	Inclusion of topics in schools and academic curricula	n/a	√	n/a
**Policy**	A	Presence of RI in committees	n/a	√	n/a
	A	Provision of databases and experts in support of public policy	n/a	√	n/a
	O	Success rate of funding grants	n/a	√	n/a
	I	Increase trust in science	n/a	√	n/a
	I	Notable changes in funding decisions	n/a	√	n/a

The indicators of “Building BioData.pt” identified in the ESFRI list corroborated the previous finding that the activities with broader impact were those dedicated to the “Support of Research Communities” ( Table 4). The detailed information for this exercise is available for consultation. ^11^ In addition, “Building BioData.pt” generated indicators for all ESFRI’s areas and covered all indicators except number 14 *(number of publicly available data sets used externally).*

**Table 4.** Indicators of “Building BioData.pt” found in the ESFRI list of indicators and impact areas. Tick – indicator or proxy found; n/a – not applicable. WG (Working Group) 1 – Plants, WG2 – Marine Resources, WG3 – Systems biology, WG4 – Neurosciences, WG5 – Yeastract, WG6 – Common Infrastructure, WG7 – Training, WG8 – Industry & Entrepreneurship, WG9 – Bioinformatics Support and WG10 – Project Management & Dissemination.

Impact area	BioData.pt Infrastructure Objectives	Strengthening research, technological development and innovation		Enhancing research knowledge transfer from academy to industry
	BioData.pt Project Working Groups	Research Communities (WG1, 2, 3, 4, 5)	Support to Research Communities (WG6, 7, 9, 10)	Industry & Entrepreneurship (WG8)
**Achieving scientific excellence**	1. Number of user requests for access	n/a	√	n/a
	2. Number of users served	√	√	n/a
	3. Number of publications	√	n/a	n/a
	4. Percentage of top (10%) cited publications	√	n/a	n/a
**Delivery of education and training**	5. Number of MSc and PhD students using the RI	n/a	√	n/a
	6. Training of people who are not RI staff	√	√	n/a
**Enhancing collaboration in Europe**	7. Number of Members of the RI from ESFRI countries	n/a	√	n/a
	8. Share of users and publications per ESFRI member country	√	n/a	n/a
**Facilitating economic activities**	9. Share of users associated with industry and publications with industry	n/a	n/a	√
	10. Income from commercial activities and the number of entities paying for service	n/a	√	n/a
**Outreach to the public**	11. Engagement achieved by direct contact	√	√	√
	12. Outreach through media	n/a	√	n/a
	13. Outreach via the RI’s own web and social media	n/a	√	n/a
**Provision of scientific advice**	15. Participation by RIs in policy related activities	n/a	√	n/a
	16. Citations in policy related publications	n/a	√	n/a
**Facilitating international co-operation**	17. Share of users and publications per non-ESFRI member country	√	n/a	n/a
	18. International trainees	n/a	√	n/a
	19. Number of members of the RI from non-ESFRI countries	n/a	√	n/a
**Optimising management**	20. Revenues	n/a	√	n/a
	21. Extent of resources made available	√	n/a	n/a

The analysis of “Building BioData.pt” indicators using the OECD framework ( Table 5) also corroborated the broad impact of the project, by retrieving indicators for all impact areas. In this case, the scientific impact produced by the activities of “Research Communities” assumes particular relevance. Nonetheless, as for the previous frameworks, the activities related to “Support to Research Communities” have a wider impact. The full dataset of this analysis can be consulted. ^11^

**Table 5.** “Building BioData.pt” indicators found in the OECD list of indicators and impact areas. Tick - indicator or proxy found; n/a - not applicable. WG (Working Group) 1 - Plants, WG2 - Marine Resources, WG3 - Systems biology, WG4 - Neurosciences, WG5 - Yeastract, WG6 - Common Infrastructure, WG7 - Training, WG8 - Industry & Entrepreneurship, WG9 - Bioinformatics Support and WG10 - Project Management & Dissemination.

Impact area	BioData.pt Infrastructure Objectives	Strengthening research, technological development and innovation		Enhancing research knowledge transfer from academy to industry
	BioData.pt Project Working Groups	Research Communities (WG1, 2, 3, 4, 5)	Support to Research Communities (WG6, 7, 9, 10)	Industry & Entrepreneurship (WG8)
**Scientific impact**	S1 - Number of publications	√	n/a	n/a
	S2 - Number of citations	√	n/a	n/a
	S3 - Number of publications in High-Impact factor journals	√	n/a	n/a
	S4 - Number of projects granted	n/a	√	n/a
	S6 - Number of scientific users	√	√	n/a
	S7 - User satisfaction	n/a	√	n/a
	S8 - User project excellence	n/a	√	n/a
	S9 - Collaboration excellence (scientific)	√	n/a	n/a
	S10 - Structuring effects of the RI on the scientific community	√	√	n/a
	S11 - Papers co-authored with regional universities	√		n/a
	S12 - Use and production of open data	√	n/a	n/a
	S13 - Data openness	√	√	n/a
**Technological impact**	T15 - National grants	√	√	√
	T16 - Collaboration with national industry	√	n/a	√
	T21 - Joint technology development projects between RI and industry	n/a	n/a	√
	T24 - Collaborative projects with regional industrial partners	n/a	n/a	√
	T26 - Collaborative projects with industrial partners	n/a	n/a	n/a
	T27 - Data Sharing	n/a	n/a	√
	T29 - Data usage	√	n/a	n/a
**Economic impact**	E34 - Number of full time equivalent within the RI	n/a	√	n/a
	E36 - Number of employees	n/a	√	n/a
**Training and Education impact**	H38 - Trained students satisfaction	n/a	√	n/a
	H39 - Use of the data for training	√	n/a	n/a
	H40 - Number of graduates (regional)	n/a	√	n/a
	H43 - Students trained and distribution	n/a	√	n/a
	H44 - Training programmes for high level students	n/a	√	n/a
	H45 - Educational and outreach activities	√	√	√
**Social and Societal impact**	O47 - Production of resources in support of public policies	n/a	√	n/a
	O50 - Public awareness	n/a	√	n/a
	O52 - Popularity of the RI (public and users)	n/a	√	n/a
	O53 - Knowledge sharing	√	√	n/a
	O54 - Openess to public	n/a	√	√

The indicators generated by “Industry and Entrepreneurship” activities could find matches or proxies in the lists of the three impact assessment approaches, with greater emphasis, as expected, in the Economic and Innovation impact areas, enriching the project with a translational dimension.

Overall, this analysis showed that the activities carried out to accomplish the “Building BioData.pt” overarching objectives of “Strengthening research, technological development and innovation” and “Enhancing research knowledge transfer from academy to industry” covered the broad range of impact areas foreseen for European Research Infrastructures. This not only puts this RI of the National Roadmap aligned with sibling international counterparts, but also suggests that BioData.pt is well aligned with ELIXIR, bringing to the national scientific system the state-of-the-art topics of computing, bioinformatics and data management, addressed by this ESFRI Landmark.

**Figure 3.**
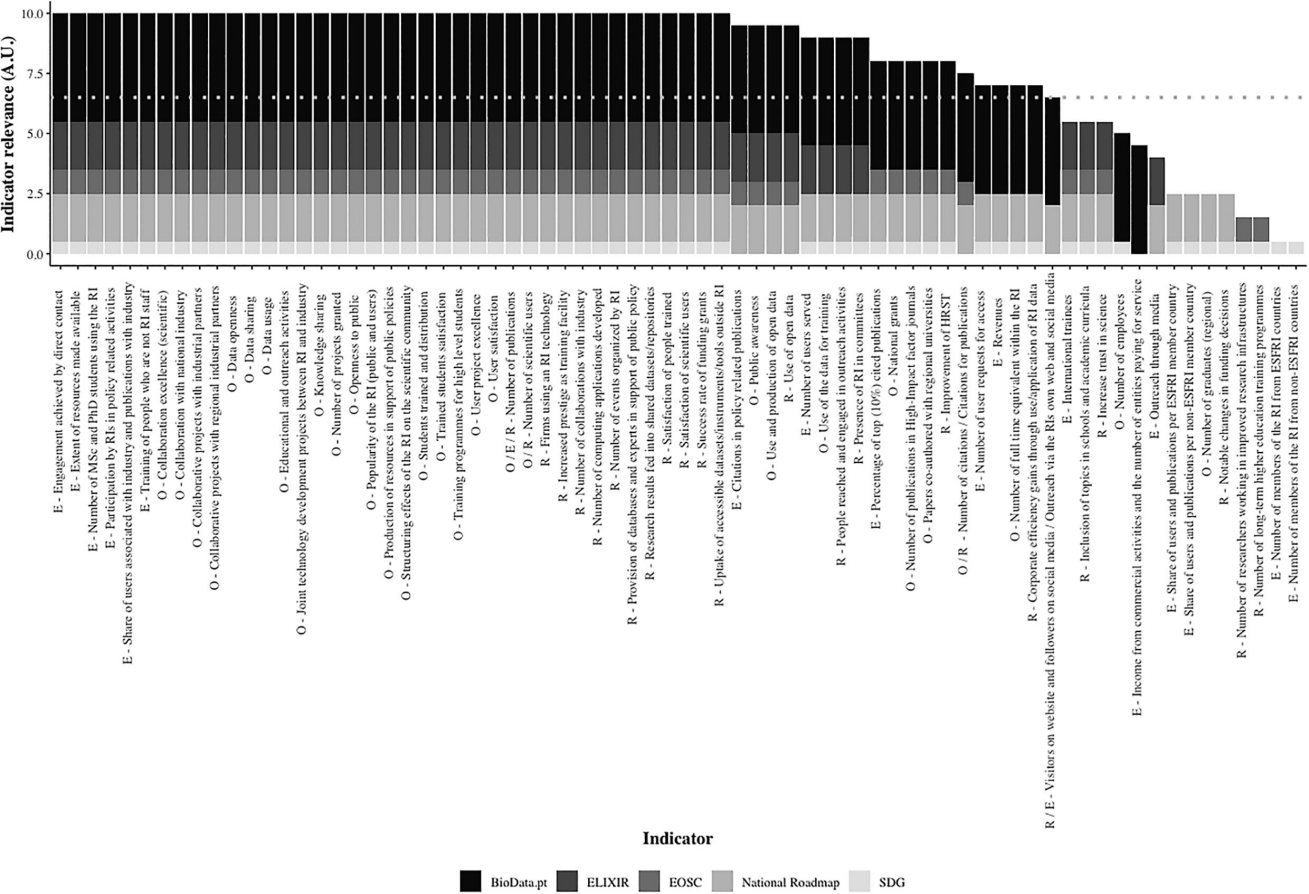
Relevance of “Building BioData.pt” indicators identified in ESFRI (E), OECD (O) and RI-PATHS (R) impact assessment frameworks. Arbitrary units (AU). Dashed line - cutoff point.

Finally, the relevance of the retrieved indicators was ascertained, considering the national context and the global environment of the BioData.pt mission. Arbitrary units (A.U.) were attributed to the alignment of each indicator with the objectives/goals of the BioData.pt (4.5 A.U.), ELIXIR (2 A.U.), National Roadmap of Research Infrastructures (2 A.U.), EOSC (1 A.U.) and the Sustainable Development Goals - SDG (0.5 A.U.). A cutoff was established at 6.5 A.U., a score that could only be reached if an indicator matched BioData.pt goals and, at least, those of ELIXIR or the National Roadmap of Research Infrastructures, to which BioData.pt is bound by a formal relationship. Alignment with EOSC and SDG was sought to assess the chosen indicators in agreement with the main European trends in open science and the goals for global development, respectively ( Figure 3). The full dataset of this analysis can be consulted. ^12^

## Discussion

Of the 102 indicators compiled as potentially relevant to RIs by the RI-PATHS project, 25 indicators were monitored during “Building BioData.pt”, scattered in all impact areas, suggesting the broad socio-economic impact of BioData.pt. RI-PATHS also proposes a set of pathways that should be taken into consideration when assessing the socio-economic impact of specific project activities. For instance, pathways 5 - *Learning and training by using RI facilities and services*, 9 - *Provision of specifically curated/edited data*, and 11 - *Creating and shaping scientific networks and communities* could be applied in the monitoring the impact of BioData.pt training activities (e.g., “ Ready for BioData Management?”), the CorkOak DB portal ^13^ and the consolidation of our Communities and Platforms, in a multidisciplinary network to identify gaps in computing data management and bioinformatics for the life sciences and build solutions. In these cases, around 50% alignment with the primary indicators of each referred pathway was observed, while only 25% alignment was detected when all BioData.pt project indicators were compared with the overall indicators list. These findings emphasize the need to adjust global performance and impact indicators to the particular context of a national project (e.g., with a wide scope that covered simultaneously *Enabling Science*, *Problem-Solving,* and *Science and Society*, in the case of the establishment of a national RI) or to a particular activity with a narrower scope (e.g., a training program, a community, a data portal).

Of the 21 indicators compiled by the ESFRI framework, 20 were monitored by “Building BioData.pt”. The missing indicator, *Number of publicly available data sets used externally*, relates to the optimisation of data use and is a relevant indicator for a RI like BioData.pt. Although BioData.pt has publicly available datasets ( *e.g.*, Pheno and CorkOakDB ^13^), it does not currently maintain indicators to demonstrate how these are being used externally. This difficulty also intricates with the fact that there is still a long way to go in the formal acknowledgement of RI services by its users, which is harder when the goods provided are intangible and sometimes provided by third parties. In the future, this topic should be addressed by putting in place a tracking system.

Finally, out of the 58 indicators compiled by the OECD framework, 32 were monitored by “Building BioData.pt”, 18 belonging to Scientific Impact and Training and Education, emphasizing the importance of these areas into BioData.pt.

The three frameworks considered in this study were comprehensive enough to accommodate all indicators maintained during “Building BioData.pt”, in spite of the fact that these were tailored according, for instance, to its specific national context, mission and role as a national node of a European RI. ^14^ In addition, considering absent indicators may also prove useful to bring our attention to untracked activities that are relevant to the mission of a RI.

The vast majority of the ESFRI, OECD and RI-PATHS indicators identified in Building BioData.pt surpassed the 6.5 A.U. in our scoring exercise, indicating that, this project was well aligned with current thinking around the performance and impact of RIs. This also reflects the natural integration of BioData.pt in the European and Portuguese research landscape. Furthermore, alignment with the EOSC goals was also very frequently observed underlining the commitment of BioData.pt with the best practices of FAIR data management and open science. It was interesting to observe the overlap between BioData.pt and SDG objectives, suggesting that national RIs, as likely their European counterparts, can give a valuable contribution to global sustainable development, and are thus a public good. Below the cutoff, three indicators contributing to the strategic objectives of the BioData.pt infrastructure were identified that did not align with the priorities of ELIXIR or the National RoadMap of Research Infrastructures. These were the *number of employees* and the *income from commercial activities and the number of entities paying* for service. Although these indicators are not directly related to the mission of BioData.pt, they may assume a very relevant role for its long-term sustainability and thus, perhaps both the National Roadmap for Research Infrastructures and ELIXIR would like to consider this information when revising their priorities.

## Final remarks

The monitoring of RIs performance and impact through indicators can be powerful in strategic management not only for continuous monitoring but also to inform future steps at the level of funders, optimization of processes and visibility by users and citizens. ^15^^,^^16^ Although highly qualified consultants are available to provide such service, national RIs are typically underfunded, and thus cannot support the cost. It is thus critical to establish simple and participatory methods to be implemented by the RI staff with oversight from the management team.

From this study, it is clear that the approaches developed by ESFRI, OECD and RI-PATHS are very useful and adaptable to the national context. It is also clear that special attention should be given to the national and thematic context of the activities under assessment.

Overall, there is a strong alignment of “Building BioData.pt” with the mission of a research infrastructure as foreseen by ESFRI. The major flaw recognised under this study, respecting the difficulty to control external use and acknowledgement of RI assets, such us datasets, may need a collective solution to be addressed in the future.

This exercise was done backwards to provide lessons for the future, where *ex ante* the areas where impact aims to be achieved, the different types indicators for each activity to be collected, as well as the methods for capturing should be documented.

In this scope, the methodology used to assess the relevance of the indicators maintained during “Building BioData.pt” ( Figure 3) can be useful to adapt the selection of indicators to a specific context, taking into account the mission and goals of the RI, and also those of national and international organizations and global initiatives composing the landscape where the RI develops its work and aims to have impact. To successfully assess performance and impact of RIs it is also very important to set up processes where the staff should be involved already from the planning stage, such as the BioData.pt monitoring and evaluation matrix ( Figure 2).

## Data availability

### Underlying data

Zenodo: Mapping Building BioData.pt Indicators against the performance and impact assessment frameworks for research infrastructures of OECD, ESFRI and RI-PATHS project, https://doi.org/10.5281/zenodo.5828310. ^11^

Zenodo: Relevance of “Building BioData.pt” indicators identified in ESFRI (E), OECD (O) and RI-PATHS (R) impact assessment frameworks, https://doi.org/10.5281/zenodo.5828295. ^12^

Data are available under the terms of the Creative Commons Attribution 4.0 International license (CC-BY 4.0).
