# Supplementary material for: Making European performance and impact assessment frameworks for research infrastructures glocal
Source: F1000Res. 2022 Aug 2;11:ELIXIR-278. Originally published 2022 Mar 4. [Version 2] doi: 10.12688/f1000research.108804.2 (PMC9372636; doi:10.12688/f1000research.108804.2)
Supplement: Peer review history

# Peer review: Reviewer response for version 2

**DOI:** 10.5256/f1000research.136830.r146238

**Published:** 2022-08-11

**Authors:** Enrico Guarini, Marialuisa Lavitrano

**Affiliations:** 1 Department of Business and Law, University of Milano-Bicocca, Milan, Italy; 2 School of Medicine and Surgery, University of Milano-Bicocca, Milan, Italy; 3 BBMRI-ERIC (Biobanking and Biomolecular Resources Research Infrastructure), Graz, Austria; 4 EOSC (European Open Science Cloud Association), Brussels, Belgium

The authors have adequately addressed all the issues that had been raised in our review report. The result is an interesting paper on a very relevant topic for the management of research infrastructures. We have no more comments, and we recommend indexing. Thank you for inviting us in reviewing this paper.

Is the topic of the opinion article discussed accurately in the context of the current literature?

Yes

Are arguments sufficiently supported by evidence from the published literature?

Yes

Are all factual statements correct and adequately supported by citations?

Yes

Are the conclusions drawn balanced and justified on the basis of the presented arguments?

Partly

Reviewer Expertise:

Financial management and governance in government and not-for-profit settings; Performance Management; Management of Research Infrastructures

We confirm that we have read this submission and believe that we have an appropriate level of expertise to confirm that it is of an acceptable scientific standard.

# Peer review: Reviewer response for version 1

**DOI:** 10.5256/f1000research.120233.r126307

**Published:** 2022-04-06

**Authors:** Enrico Guarini, Marialuisa Lavitrano

**Affiliations:** 1 Department of Business and Law, University of Milano-Bicocca, Milan, Italy; 2 School of Medicine and Surgery, University of Milano-Bicocca, Milan, Italy; 3 BBMRI-ERIC (Biobanking and Biomolecular Resources Research Infrastructure), Graz, Austria; 4 EOSC (European Open Science Cloud Association), Brussels, Belgium

This paper examines how the international frameworks developed for the monitoring and evaluation of performance and impact of research infrastructures (RIs) can be adopted by national RIs to support its management. This is done by presenting the results of a four-year project of collecting and analyzing performance indicators carried out during the development of the Portuguese Infrastructure of Biological Data (BioData.pt) as the national node of an international RI (ELIXIR). The authors focus on three performance measurement frameworks developed for RIs (ESFRI, OECD, and RI-PATHS) and empirically assess how the combination of suggested performance indicators can be applied at the operational level to assess the impact of the organization. Within this perspective, the authors aim to contribute to developing useful tools for RI management.

 The article is clear and well written, however, it needs improvement. Our main point is that the authors could better discuss the management implications of their findings.

 Below we present our main points and suggestions: 

- Firstly, the authors focus from the beginning on the process of development of the national node - the ‘Building BioData.pt’ project - paying scarce attention in the Discussion section to the operational phase, for example, which indicators can also be relevant for the permanent organization/node itself rather than for the project phases. We would encourage the authors to clarify this from the beginning since the abstract seems to propose general implications for RI management. We would like to see some reflections at a general level (maybe also in the conclusions).
- One major point that needs further specification is, first of all, the adaptability of the three frameworks at the national node level. The authors present a list of 77 retrieved indicators out of 181 from the three frameworks. It is still not clear why some indicators were more relevant than others, and why many of the proposed indicators were excluded. The authors need to better clarify if this was related to the need to adjust the impact indicators to the context of the project, to the construction stage of the RI, to the national goals/strategy, or to data availability. Thus, the authors need to discuss in more detail how the case studied and the findings might affect the generalisability of the results. In other words, the issue of adaptation from ‘global’ to ‘local’ needs to be better addressed (in the discussion/conclusion).
- Another point that requires further specification is not only the relation between the indicators and the national context/strategy but also the managerial implications of collecting and using these indicators. Apart from the issue of collecting data from third parties, we think that the process of staff engagement in the measurement process should be discussed further. For example, the authors could consider the opportunity to provide more insights into how the participatory approach was conducted and received by the staff. The engagement of team members in performance measurement could be an important aspect to be emphasised as a management implication.
- In the ‘Methods section,’ the authors report the results of scoring of indicators to assess their alignment with national and international goals. No information is provided about the method and reliability of the scoring process. Moreover, it is not clarified what is meant here with the concept of ‘alignment’.
- Since the article focuses on RIs, we would encourage the authors to include it in the title. 

Minor comments: 

- Table 1. To improve readability, we would suggest adding a label ‘Activity’ to refer to “Research Communities”, “Support to Research Communities”, and “Industry engagement”, and a label ‘Objectives’ to refer to “Strengthening research, technological development and innovation" (WGs 1, 2, 3, 4, 5, 6, 7, 9, 10), and to “Enhancing research knowledge transfer from academy to industry” (WG8).
- Table 1. WG9 is missing.
- Table 1. The activity “Industry Engagement” is mentioned as “Industry and Entrepreneurship” in the ‘Implementation and impact analysis’ section.
- Methods. Clarify the years during which the measurement process was conducted.

Is the topic of the opinion article discussed accurately in the context of the current literature?

Yes

Are arguments sufficiently supported by evidence from the published literature?

Yes

Are all factual statements correct and adequately supported by citations?

Yes

Are the conclusions drawn balanced and justified on the basis of the presented arguments?

Partly

Reviewer Expertise:

Financial management and governance in government and not-for-profit settings; Performance Management; Management of Research Infrastructures

We confirm that we have read this submission and believe that we have an appropriate level of expertise to confirm that it is of an acceptable scientific standard, however we have significant reservations, as outlined above.

# Response

**Published:** 2022-07-28

**Authors:** Ana Melo

**Affiliations:** BioData.pt - Portuguese Infrastructure of Biological Data, Portugal

Dear Drs Lavitrano and Guarini,

 The authors have read thoroughly read your appreciation of this article and proceeded to the necessary modifications. We are very grateful for your analysis and think that it helped us to improve the quality of the article.

 In detail:

- Firstly, the authors focus from the beginning on the process of development of the national node - the ‘Building BioData.pt’ project - paying scarce attention in the Discussion section to the operational phase, for example, which indicators can also be relevant for the permanent organization/node itself rather than for the project phases. We would encourage the authors to clarify this from the beginning since the abstract seems to propose general implications for RI management. We would like to see some reflections at a general level (maybe also in the conclusions).

A paragraph was added to the discussion to suggest a set of indicators that can be relevant for the operational stage of BioData.pt, as a mature infrastructure.

- One major point that needs further specification is, first of all, the adaptability of the three frameworks at the national node level. The authors present a list of 77 retrieved indicators out of 181 from the three frameworks. It is still not clear why some indicators were more relevant than others, and why many of the proposed indicators were excluded. The authors need to better clarify if this was related to the need to adjust the impact indicators to the context of the project, to the construction stage of the RI, to the national goals/strategy, or to data availability. Thus, the authors need to discuss in more detail how the case studied and the findings might affect the generalisability of the results. In other words, the issue of adaptation from ‘global’ to ‘local’ needs to be better addressed (in the discussion/conclusion).

The discussion was reformulated to address and clarify the adaptability of the frameworks to the national node level, and the criteria to discriminate and attribute relevance to the indicators. Overall, the discussion section is now more detailed and emphasises the issue of adaptation from ‘global’ to ‘local’ needs.

- Another point that requires further specification is not only the relation between the indicators and the national context/strategy but also the managerial implications of collecting and using these indicators. Apart from the issue of collecting data from third parties, we think that the process of staff engagement in the measurement process should be discussed further. For example, the authors could consider the opportunity to provide more insights into how the participatory approach was conducted and received by the staff. The engagement of team members in performance measurement could be an important aspect to be emphasised as a management implication.

The managerial implications of using the selected indicators are described, and the process of staff engagement is documented including recommendations for future assessments.

- In the ‘Methods section,’ the authors report the results of scoring of indicators to assess their alignment with national and international goals. No information is provided about the method and reliability of the scoring process. Moreover, it is not clarified what is meant here with the concept of ‘alignment’.

The methods section was modified to clarify the concept of "alignment" and further information about the method and scoring process was included.

- Since the article focuses on RIs, we would encourage the authors to include it in the title. 

The title was modified accordingly.

 Minor comments: 

- Table 1. To improve readability, we would suggest adding a label ‘Activity’ to refer to “Research Communities”, “Support to Research Communities”, and “Industry engagement”, and a label ‘Objectives’ to refer to “Strengthening research, technological development and innovation" (WGs 1, 2, 3, 4, 5, 6, 7, 9, 10), and to “Enhancing research knowledge transfer from academy to industry” (WG8).

The table was modified accordingly.

- Table 1. WG9 is missing.

The table was modified accordingly.

- Table 1. The activity “Industry Engagement” is mentioned as “Industry and Entrepreneurship” in the ‘Implementation and impact analysis’ section.

The table was modified accordingly.

- Methods. Clarify the years during which the measurement process was conducted.

This information was added to the methods.

 We expect that now you will find the article acceptable for publication.

 Yours sincerely,

 Ana Portugal Melo

# Peer review: Reviewer response for version 1

**DOI:** 10.5256/f1000research.120233.r126306

**Published:** 2022-03-29

**Authors:** Eugénio Campos Ferreira

**Affiliations:** 1 CEB–Centre of Biological Engineering, Universidade do Minho, Braga, Portugal; 2 LABBELS - Associate Laboratory, Guimarães, Portugal

An interesting opinion article on the performance and impact of the Biodata.pt, the Portuguese node of the ESFRI landmark ELIXIR, the European Research Infrastructure for life science. A significant coverage of indicators was compiled based on 3 frameworks: 20 out of 21 ESFRI indicators, 32 out of 58 OECD, ESFRI, OECD, RI-PATHS projects, 25 out of 102 RI-PATHS project indicators.

 The main strengths of the article are related to the use of wide-spectrum frameworks created for the analysis of pan-European and global research infrastructures to a national (Portuguese) research Infrastructure, with ad hoc adaptations to reflect the national landscape and specificity of activities. The article also underlines the possibility of conducting a backward exercise able to prospect recommendations for the future, determine areas where impact aims are to be achieved, the indicators for each activity to be collected, as well as the methods for capturing should be documented.

 A few remarks:

- The title “Making European performance and impact assessment frameworks glocal” does not allow inferring the content of the typescript. Although the article is inserted in the ELIXIR gateway, I suggest that reference be made to the performance and impact of BioData.pt according to the addressed frameworks (ESFRI, OECD, RI-PATHS project).
- One last note: the quote “Not everything that counts can be counted, and not everything that can be counted, counts”, is frequently incorrectly attributed to Albert Einstein. Probably comes from a paper published in 1963 by the sociologist, William Bruce Cameron (Cullis 2017 ^1^).

Is the topic of the opinion article discussed accurately in the context of the current literature?

Yes

Are arguments sufficiently supported by evidence from the published literature?

Yes

Are all factual statements correct and adequately supported by citations?

Yes

Are the conclusions drawn balanced and justified on the basis of the presented arguments?

Yes

Reviewer Expertise:

Systems Biology; Bioinformatics; Research management and assessment

I confirm that I have read this submission and believe that I have an appropriate level of expertise to confirm that it is of an acceptable scientific standard.

## References

[1] : Not everything that can be counted counts….. *British Journal of Haematology* .2017;177(4) : 10.1111/bjh.14626 505-506 10.1111/bjh.14626 28466987

# Response

**Published:** 2022-07-28

**Authors:** Ana Melo

**Affiliations:** BioData.pt - Portuguese Infrastructure of Biological Data, Portugal

Dear Dr Ferreira, 

 Thank you for reading our article and making suggestions that contributed to its improvement.

 The measures taken to address your concerns are described below your points:

- The title “Making European performance and impact assessment frameworks glocal” does not allow inferring the content of the typescript. Although the article is inserted in the ELIXIR gateway, I suggest that reference be made to the performance and impact of BioData.pt according to the addressed frameworks (ESFRI, OECD, RI-PATHS project).

The authors have modified the title of the article.

- One last note: the quote “Not everything that counts can be counted, and not everything that can be counted, counts”, is frequently incorrectly attributed to Albert Einstein. Probably comes from a paper published in 1963 by the sociologist, William Bruce Cameron (Cullis 2017 ^1^ ).

The authors have confirmed and modified the attribution of this quote.

 Yours sincerely,

 Ana Portugal Melo
